# Towards the *in vivo* prediction of fragility fractures with Raman spectroscopy

**DOI:** 10.1002/jrs.4706

**Published:** 2015-05-12

**Authors:** Kevin Buckley, Jemma G. Kerns, Jacqueline Vinton, Panagiotis D. Gikas, Christian Smith, Anthony W. Parker, Pavel Matousek, Allen E. Goodship

**Affiliations:** ^1^ Central Laser Facility, Research Complex at HarwellSTFC Rutherford Appleton LaboratoryHarwell OxfordOX11 0FAUK; ^2^ UCL Institute of Orthopaedics and Musculoskeletal ScienceLondonHA7 4LPUK; ^3^ Royal National Orthopaedic HospitalStanmoreHA7 4LPUK

**Keywords:** SORS, bone disease, clinical investigation, medical diagnostics, *in vivo*

## Abstract

Fragility fractures, those fractures which result from low level trauma, have a large and growing socio‐economic cost in countries with aging populations. Bone‐density‐based assessment techniques are vital for identifying populations that are at higher risk of fracture, but do not have high sensitivity when it comes to identifying individuals who will go on to have their first fragility fracture. We are developing Spatially Offset Raman Spectroscopy (SORS) as a tool for retrieving chemical information from bone non‐invasively *in vivo*. Unlike X‐ray‐based techniques SORS can retrieve chemical information from both the mineral and protein phases of the bone. This may enable better discrimination between those who will or will not go on to have a fragility fracture because both phases contribute to bone's mechanical properties. In this study we analyse excised bone with Raman spectroscopy and multivariate analysis, and then attempt to look for similar Raman signals *in vivo* using SORS. We show in the excised work that on average, bone fragments from the necks of fractured femora are more mineralised (by 5–10%) than (cadaveric) non‐fractured controls, but the mineralisation distributions of the two cohorts are largely overlapped. In our *in vivo* measurements, we observe similar, but as yet statistically underpowered, differences. After the SORS data (the first SORS measurements reported of healthy and diseased human cohorts), we identify methodological developments which will be used to improve the statistical significance of future experiments and may eventually lead to more sensitive prediction of fragility fractures. © 2015 The Authors. *Journal of Raman Spectroscopy* Published by John Wiley & Sons, Ltd.

## Introduction

Fragility fractures, those fractures which result from low level trauma, have a large and growing socio‐economic cost in countries with aging populations. For example, in the UK, over 70 000 hip fractures occur annually, and the total cost of associated care is over £2 billion. The sufferers of these fractures have 10% mortality in the 30 days after the event and up to 30% mortality within a year.^1^

The bones of those considered to be at risk of a first fragility fracture are often assessed with X‐ray radiation, with a bone's density, or areal density, being predictive of its likelihood to fracture. The predictive power of X‐ray‐measured density to measure a fracture is conceptually ‘similar to the assessment of the risk of stroke by blood pressure readings. Blood pressure values are continuously distributed in the population, as is BMD (Bone Mineral Density, areal density of the bone). In the same way that a patient above a cut‐off for blood pressure is diagnosed as hypertensive, the diagnosis of osteoporosis is based on a value for BMD below a cut‐off threshold. As is the case for blood pressure, there is no threshold of BMD that discriminates absolutely between those who will or will not have a clinical event [i.e. a fracture in the case of bones].’^2^ The World Health Organisation has set the BMD threshold for the diagnosis of osteoporosis as 2.5 standard deviations below the average for control subjects who are at their peak BMD.^3^ Thus, being diagnosed with osteoporosis does not guarantee that a fracture will occur, and conversely having normal bone density is no guarantee that a fracture will not occur.^2^

In short, bone‐density‐based techniques are vital for identifying populations that are at higher risk of fracture but do not have high sensitivity when it comes to identifying individuals who will go on to have a fragility fracture (a meta‐analysis of 11 studies involving tens of thousands of participants has shown that bone‐density‐based techniques can predict ~35% of hip fractures in populations with osteopenia or osteoporosis).^4^ Considering the prevalence and cost of fragility fractures there is a great need for more effective diagnostic tools.

The present study is concerned with the development of Spatially Offset Raman Spectroscopy (SORS) as a new clinical/diagnostic tool for assessment of bone transcutaneously *in vivo*. SORS utilises non‐ionising infra‐red light and, unlike X‐ray‐based techniques, can retrieve information from both the mineral and protein phases of the bone.^5^, ^6^ It has recently been demonstrated that SORS can be used to measure pathological ratios of mineral to organic content in bone *in vivo* 7 and, because the ratio of mineral to organic can account for a large percentage of bone's mechanical properties,^8^, ^9^, ^10^ the technique may allow better discrimination between those who will or will not, go on to have a fragility fracture.

### Bone composition, osteoporosis and fragility fracture

In his 1991 review Riggs wrote that osteoporosis is an absolute decrease in the amount of bone, and that the bone that is present is normal chemically and histologically.^11^ In the years following this review, studies appeared that were in agreement with this view: ashing was used to show that cortical and cancellous bone from fractured femoral necks had similar mineral, organic and water content as controls, 12, 13 and quantitative backscattered electron imaging (qBSEi) was used to show that there was no significant difference in the bone mineralization density distribution (BMDD) between cortical bone from the iliac crest of postmenopausal osteoporotic women and controls.^14^ Thermo‐gravimetric analysis also showed that bone from fractured femoral heads was as stable as that from control bone.^13^ X‐ray diffraction of bone from fractured femoral heads showed: (1) the mineral unit cell dimensions and crystallite sizes to be the same as in control bone, (2) the mean carbonate content to be almost the same as in control bone (7.5 and 7.6%) and (3) the position of the carbonate inclusions to be the same as in control bone (i.e. they substituted for hydroxyl groups and phosphate groups in the same way).^13^

The story did not stay simple; however, in 1993 Bailey *et al.* used biochemical analysis to reveal that collagen from (OP) fractured bone had different levels of post‐translational modifications to that from control bone.^15^ Specifically, the stabilising cross‐links and the hydroxylation of the collagen were increased in fractured bone.^15^ The qBSEi picture also became more complicated; in addition to the identical BMDD finding described above, the iliac crest study reported that the bone from the OP group had a decreased calcium content.^14^ Another study of bone from fractured femoral necks and controls showed the level of mineralisation to be lower in the (OP) fractured bone,^16^ and a third study reported that bone from (OP) fractured femurs was under mineralised compared to control bone.^17^ The 2004 study was challenging the, by now ‘traditional view’, that osteoporotic fractures were the result of a reduction in bone mass, remodelling effects and alterations in the micro‐architecture.^16^

#### Vibrational spectroscopy, osteoporosis and fragility fracture

The question of whether osteoporosis and fragility fractures are associated with altered bone‐tissue composition has also been addressed with infrared and Raman spectroscopy of excised bone.^5^, ^18^, ^19^ Fourier transform infrared (FTIR) spectroscopy has been used to show that the mineral in the bone tissue from osteoporotic patients was more crystalline and the bone has a lower mineral‐to‐collagen ratio than bone from control patients.^20^, ^21^ FTIR imaging has been used to show that spectroscopic features related to collagen maturity, higher crystallinity and higher mineral‐to‐collagen ratio are associated with higher risk of fragility fractures.^22^

A 2006 Raman spectroscopy study by the Morris group, which looked at compositional differences in women with and without osteoporotic fracture, suggested that the bone was indeed chemically altered.^23^ The data showed that the carbonate‐to‐phosphate spectral band ratio was ~20% larger in bone from the iliac crests of women who had suffered fragility fractures (the standard deviations of these figures were overlapped and *p* = 0.05). It was also reported that the mineral‐to‐collagen ratio of bone from the head of a fragility‐fractured femur was ~30% greater than that from a control femur (*p* = 0.04 and 0.11 for carbonate/amide I ratio and the phosphate ν_1_/amide I ratio, respectively).^23^

### Present study

In the present study we use Raman spectroscopy and multivariate analysis to measure compositional markers in excised fragility‐fractured bone and then explore the hypothesis that similar Raman signals can be measured transcutaneously *in vivo* using SORS.

The first aim was to use Raman spectroscopy to identify spectroscopic/chemical differences in bone excised from two cohorts (one cohort who had suffered from hip fractures and one cohort of donors who had not). We used the unsupervised multivariate analysis technique, Principal Component Analysis (PCA) rather than univariate measures (e.g. the intensity ratio of different Raman bands) because it has been shown that the statistical power of univariate measures can be lessened by many experimental factors. For example, it has been reported that changes in the relative alignment of the laser polarisation and the orientation of the mineralised collagen fibrils can cause some mineral to collagen ratios to change but leave others unaffected,^24^ that freezing the samples can cause some Raman bands to change intensity more than others^25^ and that some mineral to collagen ratios can have more variance than others.^26^ Band ratios can also be affected by the presence of fats/lipids which have Raman bands that overlap with collagen Raman bands (a sample which is covered in marrow can appear to have more intense collagen bands, i.e. be less mineralised).

The second aim of the study was to apply SORS and multivariate analysis to look for the excised‐bone Raman markers transcutaneously *in vivo* (the first SORS study to compare data from healthy and diseased human cohorts). The *in vivo* study is not structured as a clinical trial but as a clinical investigation which points the way towards the development of SORS as a clinical tool for predicting fragility fractures. After presenting the results we identify methodological developments which could improve the statistical significance of our experiments and eventually lead to more sensitive prediction of fragility fractures.

## Method/Materials

### Excised bone samples and *in vivo* measurements

#### Excised bone samples

Ten femoral heads/necks were collected from elderly patients who had undergone surgery after sustaining low‐trauma intra‐capsular fragility fracture to the neck of femur (Watford General Hospital, West Hertfordshire Hospitals NHS Trust, England). Each patient gave written consent for the material to be retained an analysed. The femoral heads were retrieved *en bloc* during surgery using a surgical helical drill. After retrieval the samples were stored briefly in a fridge (4 °C) before being transported to the laboratory.

The control specimens were from ten cadaveric femora (from six individuals) that were obtained from the Vesalius Clinical Training Centre, University of Bristol, England. Five left femora and five right femora were collected (fractures associated with metastatic cancer were excluded from the study). The head/neck of each femur was removed using an AEW Thurne 350 band saw (AEW Delford Systems, Norwich, England) with a cutting speed of 27 m/s.

**Figure 1:**
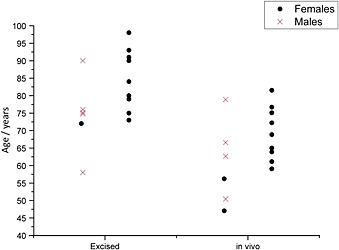
The age and gender of the excised‐bone donors and the *in vivo* patients. In each group the ‘controls’ are on the left and the ‘diseased’ are on the right. The males are plotted as crosses and the females as circles (two circles overlap at 75 years in the ‘diseased’ ‐*in vivo* group).

The cortical density of the OP samples (as measured with pQCT) was 810 mg cm^−1^ (std. dev. 37 mg cm^−1^) and the cortical density of the control samples was 910 mg cm^−1^ (std. dev. 48 mg cm^−1^); the age and gender of both the OP and the control donors are given in Fig. 1.

Scalpels were used to the remove the soft tissue from every sample (fractured and control); a 10‐mm slice was cut through the middle of each specimen in the coronal plane, using the band saw described above. The coronal slices were then washed and rinsed with deionised water to remove all visible blood and lipid. All samples were stored in a freezer (−80 °C) when not in use.

#### *In vivo* measurements

Ten patients, being treated for osteoporosis at the Royal National Orthopaedic Hospital in London, were recruited into the study. The patients all had *T*‐scores of <−2.5 in either their hip or their spine and were clinically diagnosed as osteoporotic. On average the cohort had a (left) hip *T*‐score of −2.4 (std. dev. 0.6) and a spine *T*‐score of −2.4 (std. dev. 1.2). They were all receiving bisphosphonate treatment for osteoporosis (zolendronate (*n* = 9), alendronate (*n* = 1)), and all but three participants had had at least one fragility fracture. Six controls, patients with no history of bone disease or fragility fractures, were also recruited into the study at the Royal National Orthopaedic Hospital in London. The ages and genders of the sixteen *in vivo* subjects are shown in Fig. 1. All participants were non‐smokers.

### Data collection

#### Excised bone samples

The Raman spectra were collected with a Renishaw ‘*inVia*’ Raman microscope (Renishaw plc., Gloucestershire, England). The instrument utilised a 300‐mW, 830‐nm laser and a Leica microscope with an Olympus 50×/0.5 long working distance objective lens; the optical setup resulted in losses of ~50% (i.e. laser power at the sample was ~150 mW). The laser power at the sample was ~10 mW. Each spectrum was collected for 90 s (3 × 30 s accumulations), and a minimum of fifteen spectra were taken from the cortical bone of each femoral neck (the superior and inferior cortices were probed). The samples were allowed to thaw at room temperature for a minimum of 60 min before any spectra were collected. The spectra were not collected from areas of bone that were within 2 mm of the fracture line, visible damage or cracks.^23^

#### *In vivo* measurements

The SORS instrument used to collect the *in vivo* Raman spectra was custom built by Cobalt Light Systems Ltd (Oxfordshire, England). It also utilised an 830‐nm near infrared laser. For the zero offset spectra, the laser illumination point (1‐mm spot size) and the collection point were co‐incident, and the laser power was 30 mW. For the SORS spectra the light was delivered as a ring (~1 mm thick) with variable radius (spatial offset) of up to 10 mm, the collection point was the centre of the ring.^27^ For the SORS measurements the laser power in the ring was capped at 30 mW per 3.5‐mm diameter aperture (as per BS EN 60825‐1:2007, the safety standard relevant to laser light on skin). Therefore the laser power when the beam was a spot (and less than 3.5 mm across) was 30 mW, but when it was a 1‐mm‐thick ring with radius 8 mm it was approximately 150 mW, i.e. the power automatically increased with increasing illumination area but did not exceed the safety limit.

The collected Raman light was transmitted through a fibre‐optic bundle into a spectrograph with a charge‐coupled device (CCD—Andor iDus 420 BR‐DD) detector at its output. To aid overall collection efficiency, the (low OH content CeramOptec, NA = 0.28) fibre bundle used a round configuration of 33 200‐µm‐diameter fibres to collect the filtered Raman signal; the spectrograph end of the fibre bundle was configured as a linear array to fill both the spectrograph input slit and the available vertical extent of the CCD optimally. The detector had a spectral resolution of ~8 cm^−1^, and it automatically removed any spurious signals caused by cosmic‐ray events (by comparing successive spectra and looking for outliers).

Each patient had Raman spectra collected from the phalangeal bones in the fingers and flat anterio‐medial face of the proximal tibiae. The SORS offset at the fingers was 5 mm, and the SORS offset at the tibia was 8 mm. The accumulation time for each spectrum was 60 × 1 s.

### Data processing and analysis

#### Data processing

The broad fluorescence background was removed from each spectrum (both excised and *in vivo*) using a polynomial fitting routine^28^ (script written in‐house on MATLAB—The Mathworks Inc., version 2013a). The excised spectra were then normalised to the intensity of the phosphate ν_1_ band (Microsoft Office Excel).

#### Principal component analysis—excised bone

First, the excised‐bone spectra were trimmed in order to focus on the spectral region that contained the collagen bands and a reference mineral band (the carbonate band). They were then normalised to the intensity of the carbonate mineral band and analysed using PCA with scripts that were written for MATLAB.^29^, ^30^

Principal component analysis (PCA) decomposes the data matrix into orthogonal factors and ranks them in order of variance; when the excised spectra were decomposed, it was clear that the first (main) eigenvector could be used as a measure of collagen content (eigenvectors still represent a linear combination of the true spectra and thus are not usually interpreted as pure component information but the appearance of the eigenvector (Fig. 3A) makes it clear that it is dominated by collagen). The loading, or weight on this main eigenvector, was used in the rest of the analysis/figures.

#### Decomposition of the *in vivo* spectra

The *in vivo* Raman spectra, which contained contributions from skin, lipids and bone, needed to be spectrally decomposed before they could be analysed.^6^ First, each spectrum was normalised to the intensity of the lipid band at 1299 cm^−1^, and spectra with weak mineral bands were eliminated (the phosphate ν_1_ intensity lost its shape and disappeared into other Raman‐band envelopes at ~0.45, all spectra with ν_1_ intensity below 0.45 were discounted); this resulted in a data set of at least five spectra from each individual (i.e. at least 5 min accumulation time from each individual). In our previous work^6^ we have shown that a bone spectrum can be successfully removed from *in vivo* SORS data using parallel factor analysis, but in the present study, in which we had to make do with smaller data sets, we found that the best spectral decomposition was achieved with Band Target Entropy Minimisation (BTEM).^31^, ^32^, ^33^

The BTEM decomposition was performed using a software routine that was written in‐house; the data from each individual patient was combined into a file = ‘X, Y_1_, Y_2_, Y_3_…Y_~10_’ (because a BTEM analysis was used to obtain a single ‘bone’ spectrum from each person individually the algorithm saw/removed no variance related to health‐disease, age, gender, etc.)_._ The BTEM algorithm (which also had a non‐negativity term) targeted the ν_1_ phosphate band (959–963 cm^−1^) and operated on the first five eigenvectors (‘the first few vectors are associated with real chemically important signals in the system and the remainder are associated primarily with the random instrumental and experimental noise’^31^).

#### Principal component analysis—*in vivo* spectra

The BTEM spectra that were obtained from each patient (shown in Fig. 5) represented estimates of pure bone spectra that could be compared with each other. Before they were compared they were processed further; first, they were spectrally trimmed in order to focus on the spectral region that contained the collagen bands and a reference mineral band (the carbonate band). The amide I band (at ~1660 cm^−1^) was also removed as the signal at this position was subject to considerably higher tissue absorption than Raman signals at lower wavenumbers (SORS photons have long migration paths; this magnifies differences between wavelength‐dependent absorption coefficients).^6^ The trimmed spectra were normalised to the intensity of the carbonate mineral band.

The data were then analysed using PCA with scripts that were written for MATLAB.^29^, ^30^ When the BTEM spectra were decomposed, it was clear that the first (main) eigenvector could be used as a measure of collagen content (as before, it is necessary to be aware that eigenvectors represent a linear combination of the true spectra and thus are not usually interpreted as pure component information but once again the appearance of the eigenvector (Fig. 6A) makes it clear that it is dominated by collagen). The loading, or weight on this main eigenvector, was used in the rest of the analysis/figures.

## Results

### Excised bone samples

**Figure 2:**
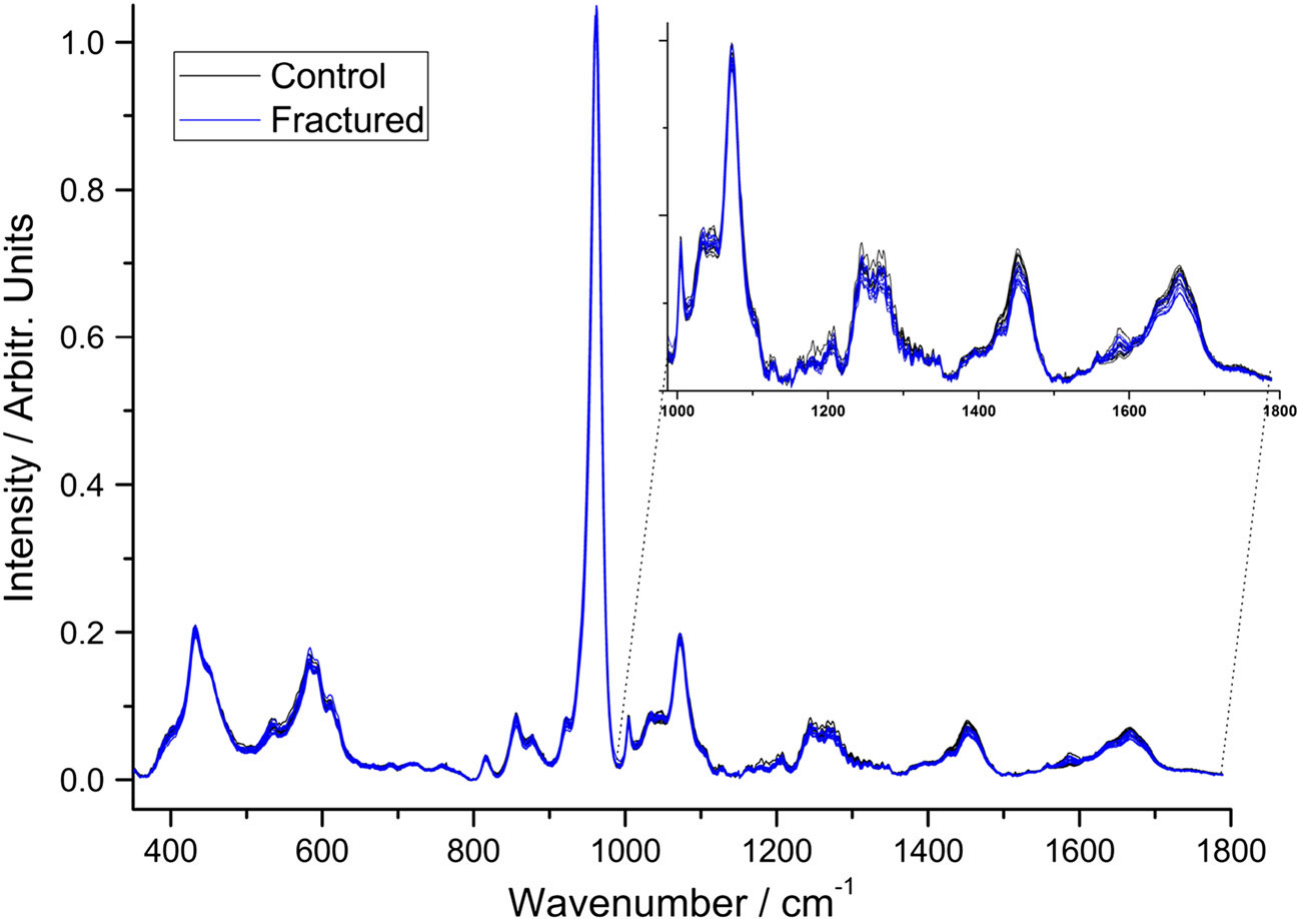
The average bone spectrum from each of the 20 excised femoral necks; they have been normalised to the dominant (ν_1_ phosphate) mineral band (10 fractured and 10 controls, *n* = 10 and *n* = 6, respectively).

The average spectrum from each of the 20 excised femoral necks (ten fractured and ten controls, *n* = 10 and *n* = 6, respectively) is shown in Fig. 2; the spectra are normalised to the ν_1_ phosphate band at 960 cm^−1^ making it apparent that the collagen bands are less intense (in relative terms) in the fractured specimens than in the control specimens.

**Figure 3:**
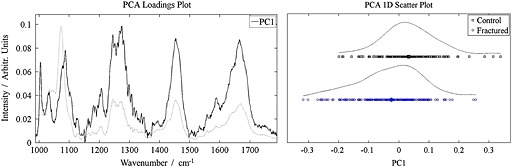
A. The reduced spectral region (from 987 cm^−1^ to 1800 cm^−1^) and the principal ‘collagen’ eigenvector associated with the excised specimens. The average spectrum is shown in the background. B. The ‘collagen score’ for each excised‐bone spectrum. The two classes are heavily overlapped.

The difference between the two sets of specimens (averages and overlaps) is shown in the multivariate analysis in Fig. 3; Fig. 3A shows the collagen eigenvector, the spectral feature which represented most of the variance in the data, and Fig. 3B shows the weighting that eigenvector has for each spectrum. There is a data point for every spectrum collected. The data points in Fig. 3B emphasise the point that the fractured femoral necks have a higher ratio of mineral‐to‐collagen than the bone from the control femoral necks (*p* < 0.0001) and that there is a large overlap between the two sets of values.

Fig. S1 shows the same data as Fig. 3B but underneath is plotted a single data point for each individual (an average of all the spectra from each person). The collapse of the intra‐individual variance upon averaging illustrates how the heterogeneity of bone on the millimetre‐scale can make it more difficult to diagnose the presence of the bone disease using point spectra.^34^ When every spectrum from each specimen is averaged the bone from the fractured femoral necks still has a significantly higher ratio of mineral to collagen than the control femoral necks (*p* < 0.02), but there is still considerable overlap between the two cohorts.

**Figure 4:**
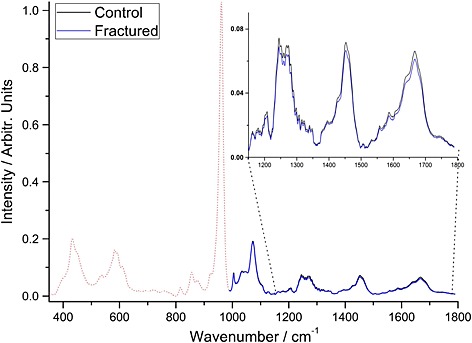
The average spectrum from the excised control bones and the average spectrum from the excised fractured bone (normalised to the carbonate/mineral band); they have been reconstructed using the information from Fig. 3. The other bands (below 1000 cm^−1^) which were not used for the reconstruction are shown for illustration.

The average Raman spectrum for the control and fractured bone specimens can be reconstructed from the data from Fig. 3, and the difference in mineralisation can be visualised (Fig. 4). The reconstructed spectra reveal that the average fractured femoral neck bone is 5–10% more mineralised (depending on which mineral/collagen bands are used in the calculation, it is 5.5% if the whole collagen region 988–1800 cm^−1^ is used). Univariate analyses of the Raman spectra, which illustrate how using different Raman bands can affect the result, are also shown in the supplementary data (Fig. S2 and Table S1).

### *In vivo* measurements

**Figure 5:**
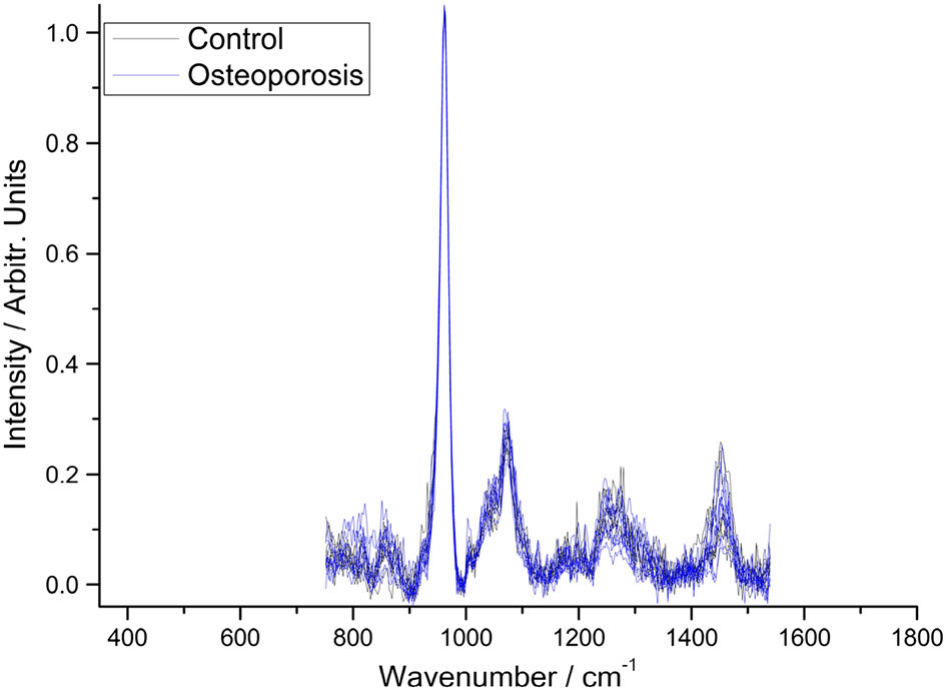
The BTEM spectrum retrieved transcutaneously from each of the 16 subjects scanned (10 osteoporotic and 6 controls).

The BTEM‐decomposed spectra retrieved from each of the 16 subjects scanned (10 osteoporotic and 6 controls) are shown in Fig. 5.

**Figure 6:**
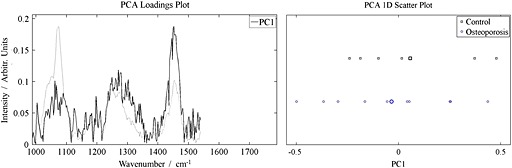
A. The reduced spectral region (from 987 cm^−1^ to 1540 cm^−1^) and the principal ‘collagen’ eigenvector for the *in vivo* measurements. The average spectrum is shown in the background. B. The ‘collagen score’ for each spectrum. The two classes are heavily overlapped.

Figure 6A shows the dominant eigenvector, and Fig. 6B shows weighting its score for each BTEM spectrum (i.e. one spectrum for each individual). The OP BTEM‐spectra have, on average, greater mineral‐to‐collagen ratios than the control BTEM‐spectra (in agreement with the excised bone data), but the difference between the two populations is not statistically significant (*p* = 0.56).

**Figure 7:**
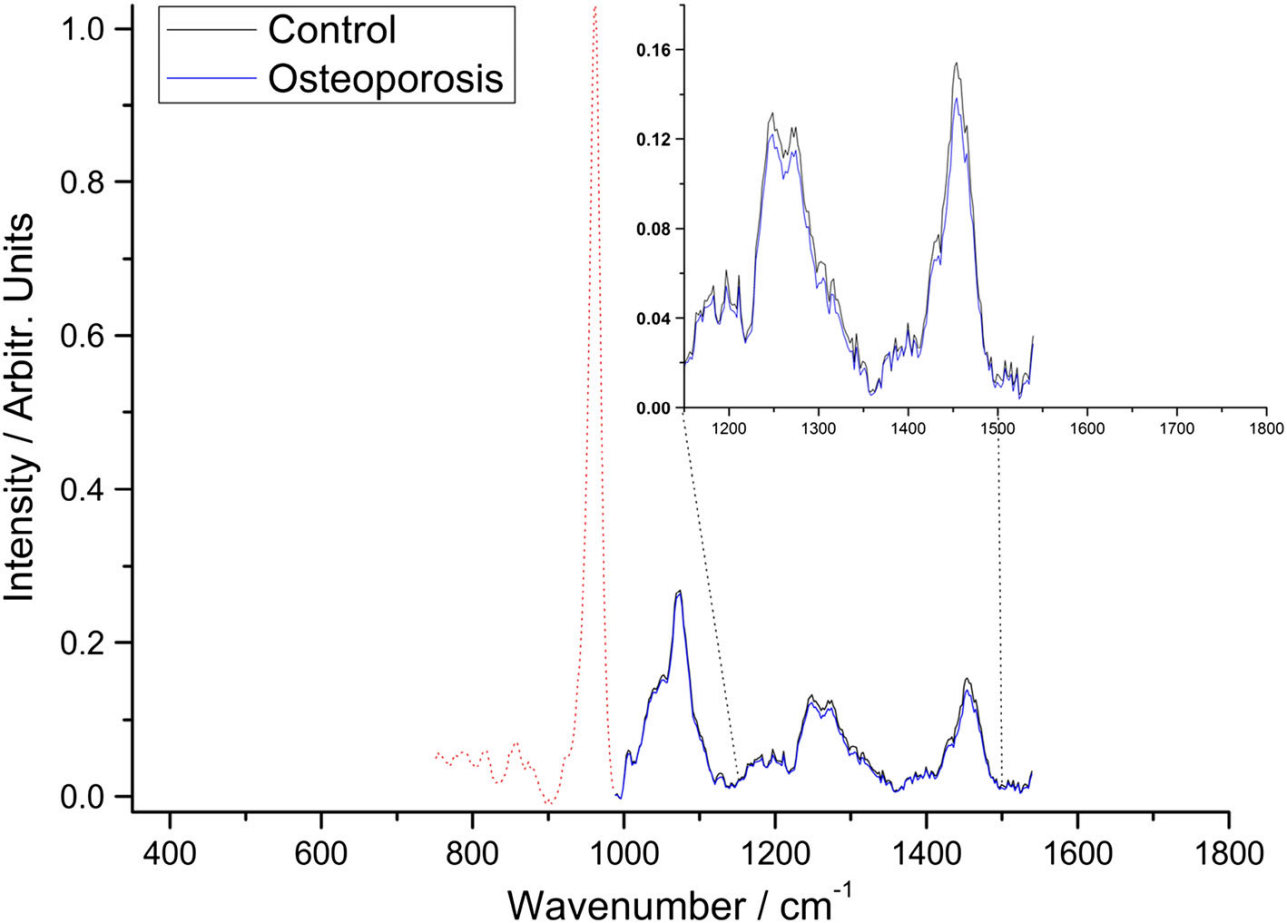
*In vivo* measurements: the average control and average osteoporosis spectra that have been reconstructed using the information from Fig. 6. The other bands (below 1000 cm^−1^) which were not used for the reconstruction are shown for illustration.

When average spectra are reconstructed for the control and fractured bone specimens using the information in Fig. 6, the difference in mineral‐to‐collagen ratio can be visualised (Fig. 7). It is also possible to use this information to calculate that spectra from a minimum of 116 people (i.e. 58 osteoporotic and 58 controls) would be required to test whether the two populations are different (to strength of *p* = 0.05).

## Discussion

Raman spectroscopy and multivariate analysis have been used to show that bone fragments from the fractured femoral necks are more mineralised (by 5–10%, *p* < 0.02) than the bone from the non‐fractured controls, but there is a large overlap between the mineralisation distributions. One can calculate how well one could predict a fragility fracture of the hip if one had access to the averaged spectra by taking the mean and standard deviations of the two groups and assuming that mineral‐to‐collagen ratios in groups have normal distributions. If we set the specificity at 90% (which is higher than the specificity of bone‐density‐based techniques when predicting hip fractures in populations with osteopenia or osteoporosis)^4^ then the averaged‐excised Raman data would predict more than 75% of hip fractures, a sensitivity more than twice that of bone‐density‐based techniques.^4^

The combination of SORS, BTEM and PCA has been used to measure bone composition of cohorts of healthy and osteoporosis individuals *in vivo*. The comparative differences we detected between the osteoporosis cohort and healthy cohorts were in the same direction (increased mineral‐to‐collagen ratio) as that between the excised‐fractured and excised‐controls. The distance between the average spectrum from each group also exhibited a similar magnitude change (an increase of 10.0 ± 2.5% for comparable spectral regions, see Fig. 4 and Fig. 7) and the two cohorts were heavily overlapped. These results, however, did not reach statistical significance.

The observed increase in mineral‐to‐collagen ratio is in agreement with a previous study.^23^ The findings cannot be explained by reference to increase in bone remodelling (in individuals with fragility‐structures/osteoporosis) because remodelling would cause spectral changes in the opposite direction, i.e. increased remodelling would be expected to increase the proportion of new/under‐mineralised osteoid and thus decrease the mineral‐to‐collagen ratio.

### Limitations of the work and scope for improvement

The current work suffered from a number of limitations related to recruiting appropriate cohorts and securing comparative excised bone samples.

#### Patient age matching

Figure 1 illustrates the age differences between the experimental groups and the control groups in this study; the donors of the excised‐fracture bones were on average 11 years older than the cadaveric donors of the excised control‐bone (85 *vs* 74), and the *in vivo*‐osteoporosis patients were on average 9 years older than the *in vivo*‐control patients (70 *vs* 61).

**Figure 8:**
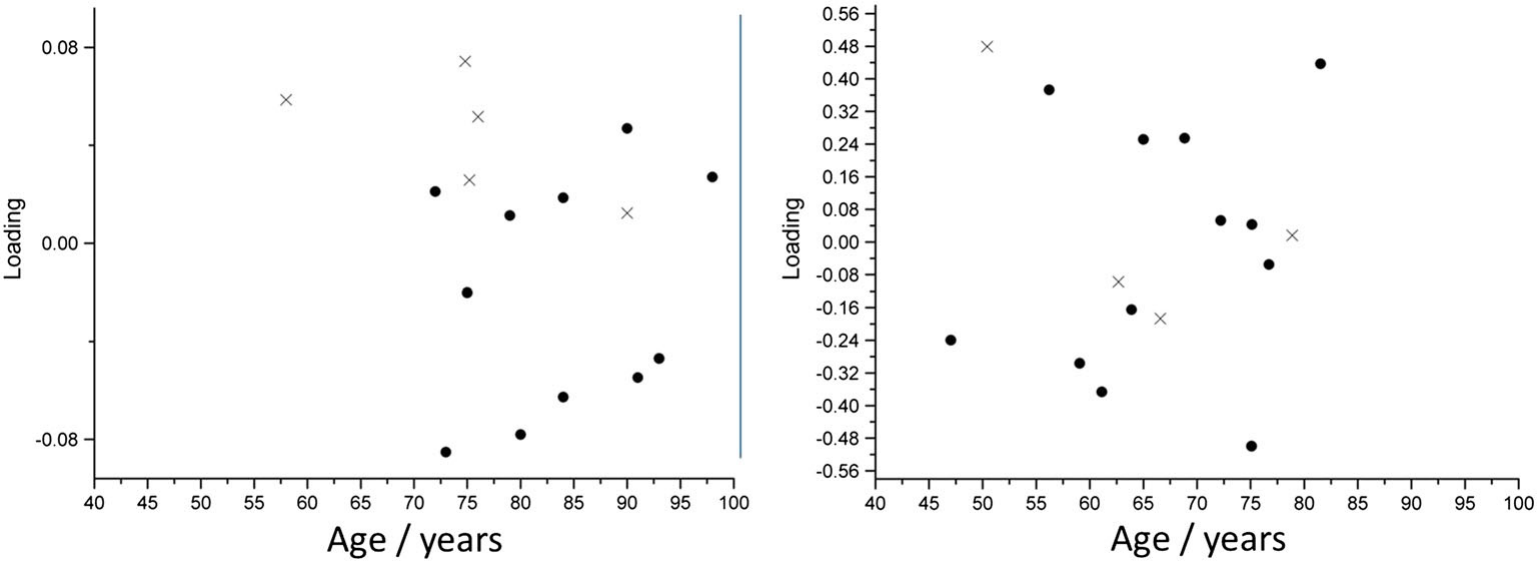
The relationship between the age (in years) *versus* the weight on the PC1 loading for the excised data (left) and the *in vivo* data (right).

There is evidence in the literature that bone mineral content varies with age^35^, ^36^, ^37^, ^38^, ^39^and that it decreases in non‐fractured individuals in the last decades of life.^40^, ^41^ We investigated the relationship between age and the collagen‐eigenvector loading in our cohorts and found no association (Fig. 8).

#### Patient gender matching

Figure 1 shows that the donors of the excised‐fracture bones (all women) were not gender matched with the cadaveric donors of the excised control‐bone (mostly men) and the *in vivo*‐osteoporotic patients (all women) were not gender‐matched with the *in vivo*‐control patients (mostly men). There is evidence in the literature that men have a higher bone mineral content than women^37^, ^38^ but our ‘more male’ groups both have lower mineral‐to‐collagen ratio than our ‘more female’ groups which suggests the gender matching does not affect the findings (there is also biomechanical evidence that at the material level bone from men is similar to bone from women^42^). We did not have enough control data to test statistically whether there was a relationship between gender and the collagen‐eigenvector, but what data we did have is plotted in supplementary Fig. S3.

#### Bisphosphonates

Bisphosphonates are a class of drug with a high affinity for the crystal surface of bone mineral and an inhibitory effect on bone resorption cells. Bisphosphonates' main action is to increase bone mass.^43^

The excised‐fractured bone samples used in this study came from people whose first presentation to clinicians was with their fragility fracture; thus, none of them had been diagnosed with osteoporosis and/or put on bisphosphonates. The excised‐control bone came to us from cadaveric donors, and we only had access to partial medical records; the bones were not visibly affected by osteoporosis or, fragility fractures but we cannot be 100% sure that none of them had been prescribed bisphosphonates during their life.

Because of the difficulty of finding osteoporotic patients who are not receiving treatment and who want to take part in a scientific investigation, all of the members of our osteoporosis‐*in vivo* cohort were taking bisphosphonates.

There is a growing body of literature on the effect bisphosphonates can have on bone composition. Studies on model systems have reported that bisphosphonates can affect how the crystals grow in bone mineral (thus affecting the size of the crystals, the degree of carbonate inclusion, etc.) and can cause a reduction in mineral‐to‐collagen ratio (in beagle dogs and ovariectomised monkeys).^43^

Vibrational spectroscopy studies of pelvic bone biopsies from humans have also shown a relationship between bisphosphonates and bone composition. In one study, which looked at bone at 0 years (baseline), 3 years and 5 years, it was shown that mineral‐to‐collagen ratio and collagen cross‐link ratio of untreated women increased with time whereas they stayed flat for women treated with risedronate (a bisphosphonate).^44^ In another study of human pelvic bone biopsies, the drugs made no difference in the crystallinity or collagen maturity but decreased the carbonate‐to‐phosphate ratio by ~15% and increased the phosphate‐to‐amide I ratio (a measure of the mineral to collagen ratio) by 20–25%.^45^ The chemical change found in our *in vivo* study of similar cohort is in the same direction, but the difference we observed is smaller (and statistically underpowered).

In another more recent study of the effects of bisphosphonate, bone biopsies were obtained from over 100 women (four cohorts: alendronate (a bisphosphonate) for 3–5 years, alendronate for >5 years, risedronate (a different bisphosphonate) for 3–5 years and risedronate for >5 years). It was shown that in strictly age‐controlled bone (i.e. the age of the bone was controlled for rather than the age of the subject) there was no difference in mineral‐to‐collagen ratio between the four cohorts.^46^

It is likely that there is no complication related to bisphosphonates in our excised‐bone data but that there is in our *in vivo* data; it will take improvements in our SORS methodology and larger cohorts of patients before we can say any more about them.

#### Anatomical‐site matching

As discussed in the introduction above there is much evidence in the literature that there is a relationship between propensity to suffer fragility fractures and bone composition (where bone composition encompasses mineral‐to‐collagen ratio, mineral composition, collagen crosslinking, etc.); it is known that bone tissue composition varies in different anatomical sites,^47^, ^48^ but it is interesting to consider whether compositional *changes* associated with fracture are localised or skeleton‐wide.

An individual is at an increased risk of fragility fracture if they have already suffered a fragility fracture in the past, for example a women who has had a vertebral fracture has ~4 times greater risk of having subsequent vertebral fractures than a women who has not had a prior fracture. More interestingly, an individual who has had one fragility fracture is about twice as likely to have a second fragility fracture at any other anatomical site as someone who has never had a fragility fracture; for example someone who has fractured their wrist is twice as likely to subsequently break their hip as someone who has never fractured a wrist.^49^ Of course, many risk factors for fragility fractures are clearly related to the whole person (e.g. cigarette smoking or propensity to fall) and so the two fractures could be considered independent events happening in the same body.^2^, ^50^

In a previous paper, we explored the regional adaptation of bone composition in the ends of long bones and showed that it had a lower mineral to collagen ratio (i.e. is less stiff) than bone found near the mid‐shaft.^48^ We discussed how any disruption to this mineralisation pattern could lead to unsuitably stiff bone being found at the proximal and distal ends of the cortex. The increase of 5–10% in the mineral to collagen ratio reported in the present study is enough to affect bone's material properties. For example, an increase of ~10% would increase the Young's modulus by ~10%.^10^ This change in material properties could also change the mechanical properties of the whole femoral neck indirectly. Computer models, in which bone deformations below a certain threshold cause resorption and bone deformations above another threshold induced bone formation, have shown that an increase in stiffness of the magnitude shown here could cause the loss of approximately 20% of the bone volume and also lead to large increases in the anisotropy of the bone architecture.^51^ Thus the patients who donated the excised‐fractured bone may have had a *localised* over‐mineralisation which affected their ability to resist fracture directly (material property changes) and indirectly (led to increased bone resorption and anisotropy).

The questions about localised changes in bone composition are related to this study because the exercised‐fracture (and exercised‐control) bone probed with conventional Raman spectroscopy was from the cortex of the femoral neck and these data were compared and contrasted with *in vivo* SORS data, which were collected from fingers and tibiae.

#### Future methodological improvements

At all stages of the *in vivo* study there were aspects that we will be able to improve as we move towards the prediction of fragility fractures transcutaneously.

- The patient recruitment: more efforts will be made to age and gender match the patients (this will dispel lingering questions about, for instance, differences in skin thickness)
- Data collection, the laser power that was used in the study was conservative and there is scope for increasing it by an order of magnitude.^52^ The throughput of the Raman collection system can also be dramatically increased by opening the spectrometer slit and using a higher dispersion grating (this is at the expense of a narrowed spectral range)^52^
- Spectral decomposition, the BTEM decomposition routine which strips away the soft tissue spectra and leaves the bone spectrum is perhaps the limiting factor in the present paper. Higher fidelity bone spectra may be achieved by accounting for tissue absorption in future analysis
- The multivariate analysis of the final (decomposed) spectra may be improved by using more sophisticated techniques, for example by combining PCA with linear discriminant analysis

Added together these measures provide scope for major improvements and may enable to deliver a method that complements or substitutes the traditional bone‐density‐based techniques.

## Conclusion

On average the bone in fractured femoral necks has a higher mineral‐to‐collagen ratio than the bone from intact controls, but there is a large overlap in the mineralisation distributions of the two groups. In our *in vivo* measurements, the first *in vivo* SORS measurements of bone in healthy and diseased human cohorts reported, we show similar but as of yet statistically underpowered differences.

## Supporting information

Supporting info item
