# Supplementary material for: Towards the in vivo prediction of fragility fractures with Raman spectroscopy
Source: J Raman Spectrosc. 2015 May 12;46(7):610–8. doi: 10.1002/jrs.4706 (PMC4976623; doi:10.1002/jrs.4706)
Supplement: Supplementary file 1 — Supporting info item [file JRS-46-610-s001.doc]

# Supplementary Material

Figure S1. The “collagen score” for each excised-bone spectrum (two top traces) plotted with the average value for each individual donor (two bottom traces).


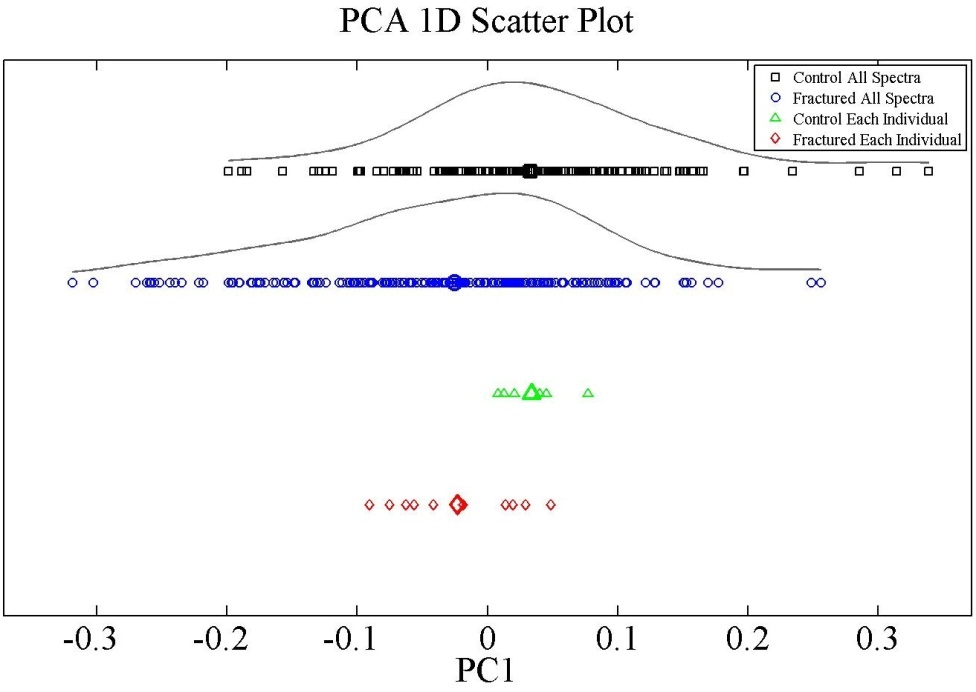


Figure S2. The average spectrum of the ten fractured femoral necks and the average spectrum of the ten controls. The residual spectrum (control - fractured) plotted below shows that in addition to the differing protein-band intensities, the main difference between the spectra comes from an apparent shift of the main phosphate band and from lipid residue on the samples (at 1300 cm^-1^, 1440  cm^-1^ & 1660  cm^-1^). It is these lipid differences that we minimised by employing multivariate analysis.


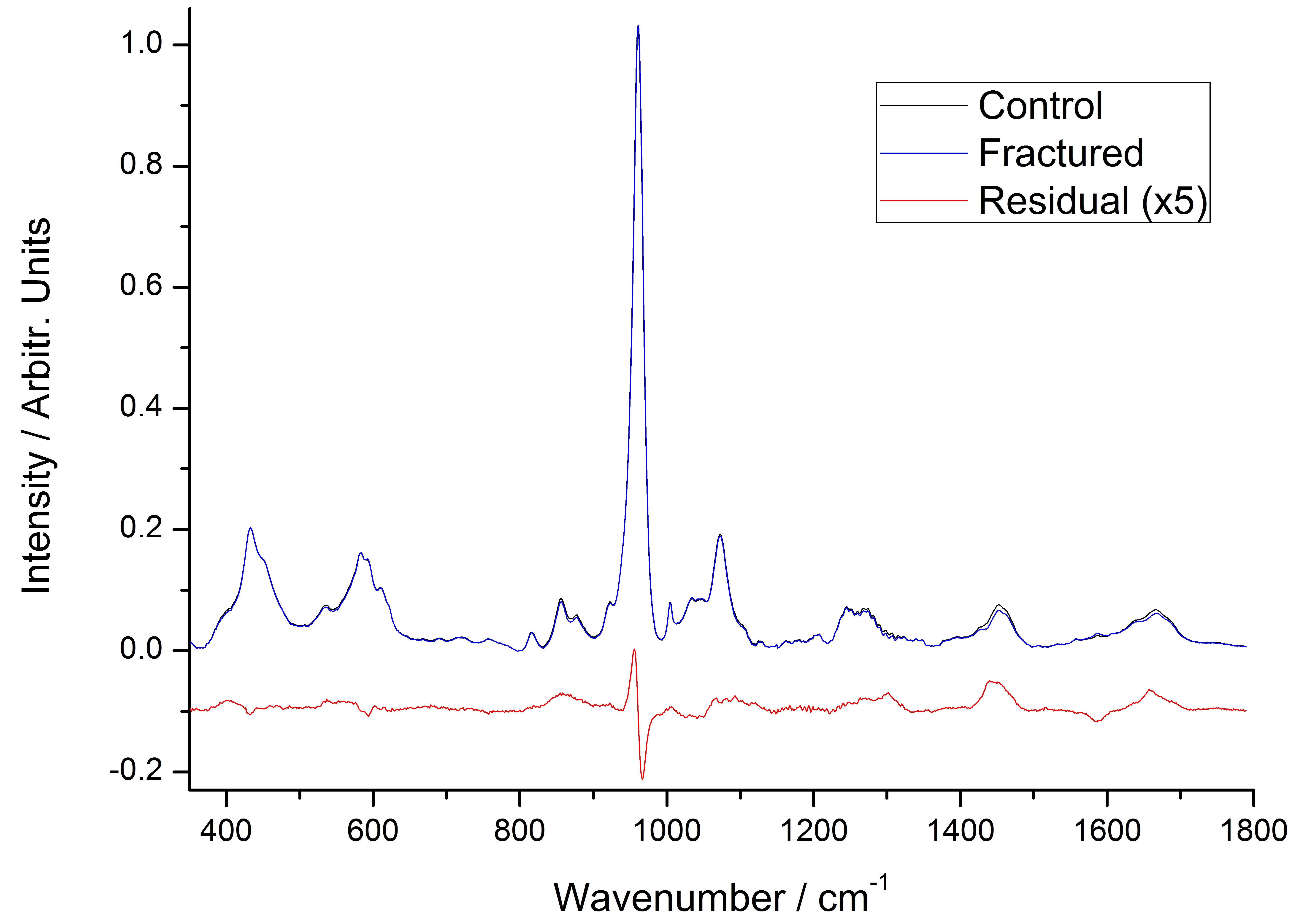


Figure S3. The relationship between the gender and PC1 loading, circles are women, crosses are men and bars are averages; Figure S3A shows the excised study and Figure S3B the *in vivo* study.


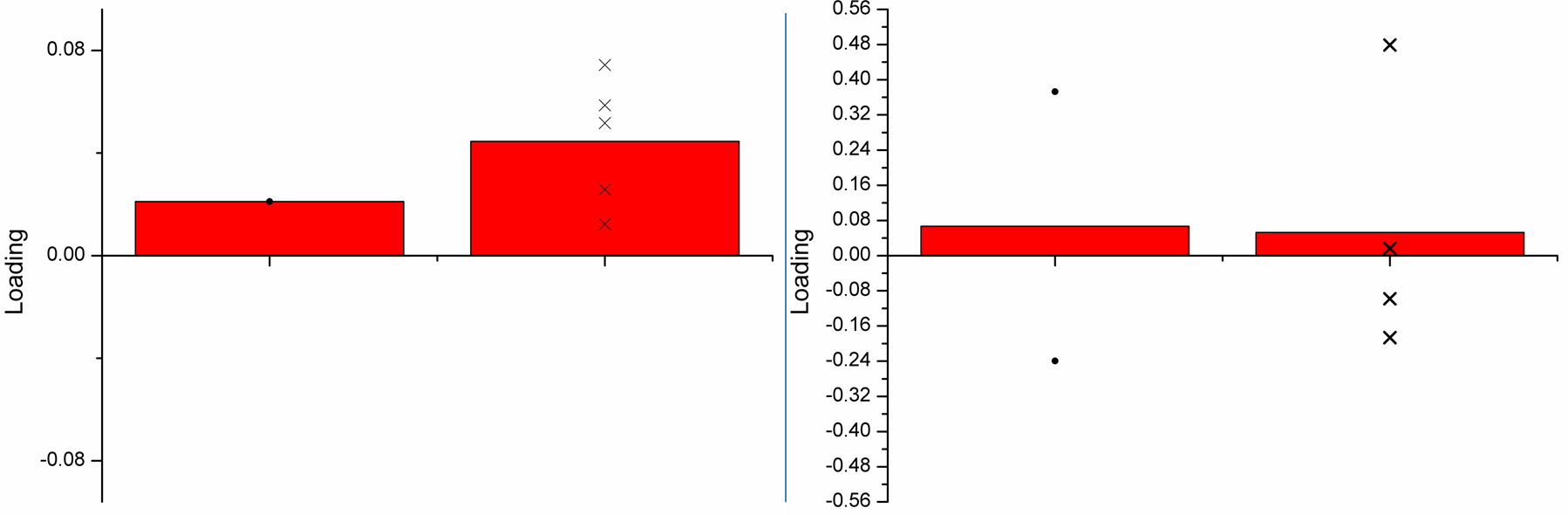


Table S1. Univariate analysis gave a similar mixed picture as that reported in Reference Error: Reference source not found. There was a statistically significant difference in mineral to collage ratio using one measure (phosphate/amide I) but a statistically weaker result using another (phosphate/amide III).

|  | Fractured | Non-fractured | **p** value |
| --- | --- | --- | --- |
| **N** | 10 | 10 |  |
| Phosphate/Carbonate | 102.23 | 100 | 0.12 |
| Phosphate/Amide I | 110.22 | 100 | 0.005 |
| Phosphate/Amide III | 105.95 | 100 | 0.078 |
